# A Multicenter Randomized Controlled Trial Comparing Single-Row With Double-Row Fixation in Arthroscopic Rotator Cuff Repair: Long-Term Follow-up

**DOI:** 10.1177/03635465211029029

**Published:** 2021-08-16

**Authors:** Peter Lapner, Ang Li, J. W. Pollock, Tinghua Zhang, Katie McIlquham, Sheila McRae, Peter MacDonald

**Affiliations:** † Division of Orthopaedic Surgery, The Ottawa Hospital, University of Ottawa, Ottawa, Ontario, Canada; ‡ Clinical Epidemiology Program, Ottawa Hospital Research Institute, University of Ottawa, Ottawa, Ontario, Canada; § Section of Orthopaedic Surgery, University of Manitoba, Winnipeg, Manitoba, Canada; ‖ Pan Am Clinic, University of Manitoba, Winnipeg, Manitoba, Canada; Investigation performed at The Ottawa Hospital, University of Ottawa, Ottawa, Ontario, Canada, and Pan Am Clinic, University of Manitoba, Winnipeg, Manitoba, Canada

**Keywords:** shoulder joint, arthroscopic surgery, rotator cuff, rotator cuff repair, single row, double row, randomized, clinical trial, long term

## Abstract

**Background:**

The long-term outcomes of single- versus double-row fixation in arthroscopic rotator cuff repair are not currently known.

**Purpose:**

To compare the treatment effects of the single- versus double-row suture technique in arthroscopic rotator cuff repair of full-thickness tears at 10-year follow-up.

**Study Design:**

Randomized controlled trial; Level of evidence, 1.

**Methods:**

Patients were evaluated at 10 years postoperatively. The primary outcome measure was the Western Ontario Rotator Cuff Index (WORC). Secondary outcome measures included the American Shoulder and Elbow Surgeons (ASES) score, Constant score, strength, and incidence of revision surgery. Ultrasound was used to evaluate the rotator cuff to determine repair integrity. Statistical analyses consistent with those of the main trial were conducted.

**Results:**

Of the original 90 participants, 77 (85%) returned at a mean follow-up of 10 years. At ten year follow-up, the WORC score was higher in the double row group (79.9 [95% CI, 16.2 to 99.1]) compared with the single row group (72.9, [95% CI, 4.3 to 100]), *P* = .020. From baseline to 2 years, the mean change in WORC scores for the single-row group was –48.5 compared with −40.6 for the double-row group, with a between-group difference of −7.8 (95% CI, −20.4 to 4.7). From 2 to 10 years, the change in WORC scores for the single-row group was 11.5 compared with −0.2 for the double-row group, with a between-group difference of 11.7 (95% CI, −0.7 to 24.3). From baseline to 10 years, the mean between-group difference was 3.9 (95% CI, −7.8 to 15.6). Similarly, a decrease in ASES scores was observed between 2 and 10 years for the single-row group (9.2 [95% CI, 0.9 to 17.5]; *P* = .029), with a nonsignificant decrease in ASES scores for the double-row group (6.2 [95% CI, −3.2 to 15.6]; *P* = .195) as well as a decrease in Constant scores for both the single- (9.5 [95% CI, 1.4 to 17.5]; *P* = .020) and double-row (14.4 [95% CI, 5.6 to 23.3]; *P* = .001) groups. Overall, 3 participants developed a full-thickness tear after 2 years: 2 from the double-row group and 1 from the single-row group. One participant from each study group underwent revision surgery after the 2-year time point.

**Conclusion:**

A statistically significant (but likely not clinically important) difference in WORC scores was seen at 10-year follow-up in favor of double-row fixation. Between baseline and 10-year follow-up, a decrease in most outcome scores was observed in both the single- and the double-row groups.

**Registration:**

NCT00508183 (ClinicalTrials.gov identifier).

Arthroscopic rotator cuff repair has become a common procedure because of improvements in surgical techniques and instrumentation. With suture anchor–based fixation methods, single- and double-row fixation techniques have emerged as the most commonly used to maximize tendon healing and improve clinical outcomes.^7^ However, early failure of rotator cuff repair is still considered the most frequently observed complication, ranging between 20% and 94% of cases.^1,10,12,15^ Biomechanical studies have demonstrated that double-row constructs lead to increased loads to failure, improved contact areas and pressures, and decreased gap formation.^16,21,23,31^ A number of authors have conducted level 1 studies to evaluate the effects of single- versus double-row repair on rotator cuff healing rates as well as on quality of life outcomes with short-term follow-up.^3,4,9,11,13,19,20^ Results showed no significant differences in outcome scores between single- and double-row repair, but single-row repair exhibited significantly higher retear rates at short-term follow-up compared with double-row repair.^25^ Given that there is a paucity of research with long-term follow-up between the 2 approaches, it is necessary to determine the enduring effects of the 2 techniques to optimize clinical results.

We have previously performed a level 1 study comparing single- and double-row rotator cuff repair.^20^ This multicenter randomized controlled trial was designed to compare the 2 techniques with respect to functional outcomes by using validated outcome measures such as the Western Ontario Rotator Cuff Index (WORC), which is a disease-specific quality of life tool for rotator cuff disease, as well as the American Shoulder and Elbow Surgeons (ASES) score and the Constant score. Anatomic outcomes were assessed with magnetic resonance imaging or ultrasonography to determine postoperative healing rates.

The study demonstrated that there were no statistically significant differences between the groups in terms of the WORC, Constant, ASES, or strength scores. Furthermore, the rate of healing in the single-row group was not significantly different from that in the double-row group at 2-year follow-up. However, multivariable logistic regression analysis adjusting for age, sagittal tear size, number of anchors, and baseline outcome scores showed that a smaller initial coronal tear size and double-row fixation were associated with higher healing rates.

The original trial involved a 2-year follow-up period, which was deemed appropriate, as soft tissue healing can be considered complete by 12 months.^18,24^ However, the long-term results of single- versus double-row repair still remain unclear in the literature. Millett et al^25^ reported a mean follow-up duration of 1.9 years (23.2 months) in a meta-analysis of level 1 randomized controlled trials. Yamaguchi et al^32^ reported an average time of 2.8 years for the majority of asymptomatic patients to become symptomatic. Thus, given that rotator cuff–mediated symptoms can occur late in the disease process, long-term follow-up would allow us to detect any subsequent deterioration in clinical symptoms and elucidate whether the progression to full-thickness tears becomes symptomatic.

The primary research objective was to determine whether patients who undergo repair of the rotator cuff with an arthroscopic technique involving double-row fixation have improved disease-specific quality of life, as measured by the WORC, at 10 years postoperatively compared with patients who undergo repair involving single-row lateral fixation. Secondary research objectives included the determination of differences in functional outcomes between the 2 groups as measured by the Constant score, the ASES score, and the incidence of revision surgery. The healing rate was determined with the use of ultrasonography. It was hypothesized that double-row fixation would yield superior quality of life and functional outcomes compared with single-row lateral fixation at long-term (10 years) follow-up.

## Methods

The methodology of the trial was described in a previous article.^20^ This was a double-blinded randomized clinical trial with two 1:1 parallel groups conducted at 2 university teaching hospitals. Recruitment for the original trial occurred between June 2007 and June 2009, and final evaluations took place between November 2017 and January 2020. The inclusion criteria were identical to those of the original randomized controlled trial, and the exclusion criteria were patients who were excluded from the original study, those who withdrew from the original study, and those who were unable or unwilling to provide written informed consent. The original study used computer-generated blocked randomization with variable block sizes. Sealed opaque envelopes were used to determine group allocation, and the envelopes were opened by the circulating nurse in the operating room once eligibility was confirmed. No changes in trial methodology or treatment outcomes occurred after the initiation of the trial. Institutional review board approval was obtained. This trial was registered at www.clinicaltrials.gov (NCT00508183). There was no external funding for this trial.

### Data Collection

Eligible patients from the previous study were contacted and assessed by blinded evaluators in outpatient orthopaedic clinics (K.M., S.M.) at 2 university-affiliated teaching centers. Patients were unblinded at the final 2-year follow-up at the conclusion of the original trial. Functional outcomes were obtained. Ultrasound was used to verify the integrity of rotator cuff tendons. An independent investigator who was blinded to the patients’ assigned treatment performed imaging evaluations.

### Outcome Measures

The WORC^17^ is a disease-specific instrument that has proven to be an accurate and valid assessment of function after rotator cuff repair. Because it is specific for rotator cuff disease of the shoulder, it is highly sensitive to small but clinically significant changes in patient function. We report results of the WORC on a 100-point scale, with 100 representing a perfect score. The ASES score^30^ is a shoulder-specific assessment developed for use in all types of shoulder problems. A score of 100 points represents a perfect ASES score. The Constant score^5^ is a validated and normalized tool in comparison with disease-free patients and places greater emphasis on range of motion and strength. The European Society of Shoulder and Elbow Surgery has adopted the Constant score as a functional assessment of the shoulder. The Constant score is an overall clinical functional evaluation and is based on a 100-point scoring system calculated from a self-assessment portion that examines pain and the ability to perform tasks of daily living as well as a clinical section that tests active range of shoulder motion and strength; the higher the score, the better the outcome. Further secondary outcome measures included strength and the incidence of revision surgery.

Ultrasound was used to determine the healing rates of both techniques. Previous studies have shown an association between rotator cuff integrity after surgery and function and strength.^18^

### Statistical Analysis

Descriptive statistics were generated for all variables: mean ± SD or median (interquartile range) for continuous variables and number (percentage) for categorical variables. An intention-to-treat analysis was undertaken: all patients’ data were analyzed according to the group to which they were allocated. The independent *t* test was used to compare means, and the chi-square or Fisher exact test was used to compare percentages (healing) between the single- and double-row groups at a specific time point. A series of mixed-effects model analyses were run with 1 between-group (study group) variable and 1 within-group (time point) variable to examine the main effects as well as any interaction. These were run for the WORC, ASES score, Constant score, and strength preoperatively, at 24 months, and at final follow-up, and they were used to account for the correlation of repeated measures in the same participant over time using a compound symmetry covariance structure. The Kenward-Roger method was used to calculate degrees of freedom. Time point, study group, and the interaction between time point and study group were included in the models to calculate the difference between groups over time. Least squares means and the mean changes from baseline to 24 months and to the last follow-up in each group with 95% CIs as well as least squares means and the mean changes from baseline to 24 months and to the last follow-up between groups with 95% CIs from the models were obtained. The 5% significance level was used for all comparisons. All analyses were conducted using SAS Version 9.4 (SAS Institute).

## Results

**Figure 1.**
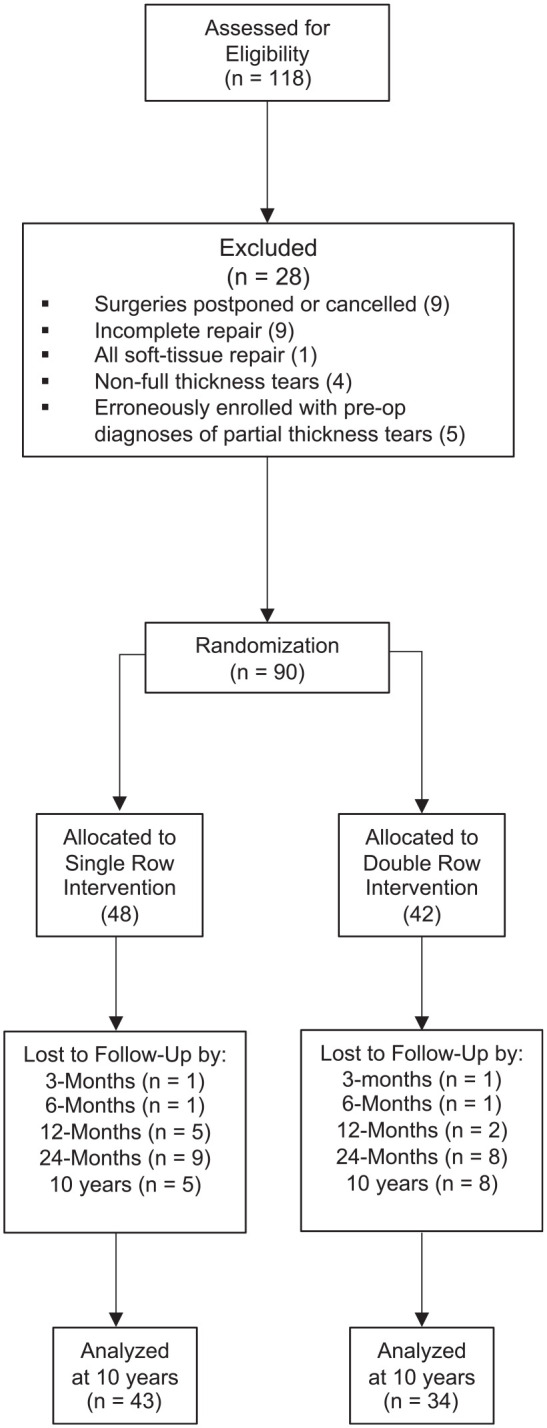
CONSORT (Consolidated Standards of Reporting Trials) diagram.

Patient flow through the study is presented in the CONSORT (Consolidated Standards of Reporting Trials) diagram (Figure 1). A total of 90 patients were randomized to undergo either single- or double-row fixation. Of these patients, 17 did not return for 24-month follow-up. At 10 years, 59 patients returned for an in-person follow-up visit, which included all outcome measures. Furthermore, 2 patients from the single-row group and 1 patient from the double-row group were not included in the analysis because of incomplete data. Overall, 33 patients in the single-row group and 28 patients in the double-row group were included in the final analysis with full outcome metrics, and an additional 16 patients agreed to complete the primary outcome measure (WORC) from home after being contacted by telephone, yielding an 85% retention rate in the trial for the primary outcome measure.

Demographic data are summarized in Table 1. Patient characteristics of those who returned at 10-year follow-up remained balanced between the groups, as was found for the entire cohort.^20^ The mean follow-up duration was 10.3 years (single-row group: 10.1 years; double-row group: 10.5 years; range, 6.9-11.0 years; *P* = .19).

**Table 1:** Baseline Demographic Data of Patients^*a*^

	All Patients			Patients With 10-y Follow-up		
	Single Row (n = 48)	Double Row (n = 42)	Total (N = 90)	Single Row (n = 43)	Double Row (n = 34)	Total (n = 77)
Age, y	56.0 ± 8.9 (38.0-82.0)	57.8 ± 7.0 (44.0-68.0)	56.8 ± 8.1 (38.0-82.8)	55.3 ± 8.2 (38.0-71.1)	57.2 ± 6.7 (44.2-68.4)	56.2 ± 7.6 (38.0-71.1)
Sex, n (%)						
Female	13 (27)	13 (31)	26 (29)	12 (28)	10 (29)	22 (29)
Male	35 (73)	29 (69)	64 (71)	31 (72)	24 (71)	55 (71)
Shoulder affected, n (%)						
Left	11 (23)	13 (31)	24 (27)	10 (23)	9 (26)	19 (25)
Right	37 (77)	29 (69)	66 (73)	33 (77)	25 (74)	58 (75)
Tear size, mm						
Coronal	21.4 ± 9.4 (10.0-41.0)	23.8 ± 10.8 (5.0-50.0)	22.5 ± 10.1 (5.0-50.0)	21.2 ± 9.1 (10.0-41.0)	22.5 ± 10.7 (5.0-50.0)	21.8 ± 9.8 (5.0-50.0)
Sagittal	18.9 ± 8.5 (8.0-45.0)	18.9 ± 6.6 (7.0-36.0)	18.9 ± 7.7 (7.0-45.0)	18.8 ± 7.4 (7.0-36.0)	18.7 ± 7.0 (8.0-40.0)	18.8 ± 7.2 (7.0-40.0)
No. of anchors, median (IQR)	1 (1-2)	2 (2-3)	2 (1-2)	1 (1-2)	2 (2-3)	2 (1-2)
Smoking status, n						
Smoker				7	7	14
Nonsmoker				36	27	63
Comcomitant biceps procedure, n (%)						
No biceps procedure				35 (81)	25 (74)	60 (78)
Biceps tenodesis				8 (19)	9 (26)	17 (22)

a Data are shown as mean ± SD (range) unless otherwise indicated. IQR, interquartile range.

**Figure 2.**
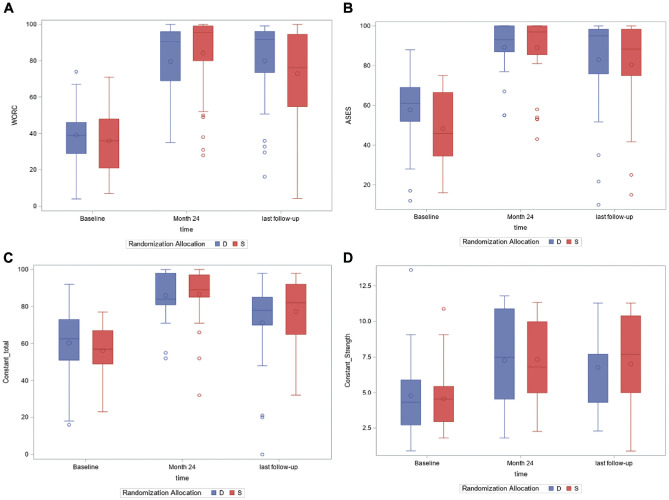
(A) Boxplot of Western Ontario Rotator Cuff Index (WORC) scores. (B) Boxplot of American Shoulder and Elbow Surgeons (ASES) scores. (C) Boxplot of Constant scores. (D) Boxplot of strength scores (kg). D, double row; S, single row.

At 10-year follow-up, the WORC scores were significantly higher in the double-row group (79.9 [95% CI, 16.2-99.1]) compared with the single-row group (72.9 [95% CI, 4.3-100.0]) (*P* = .020). No other significant differences between the single- and double-row groups were found for the WORC score at other time points or for the ASES score, Constant score, and strength at any time point (Table 2 and Figure 2).

**Table 2:** Outcome Scores by Time Point^*a*^

	Single Row (n = 48)	Double Row (n = 42)	Total (N = 90)	*P* Value
WORC				
Baseline	36.0 ± 17.2 (7.0-71.0)	39.0 ± 16.1 (4.0-74.0)	37.4 ± 16.6 (4.0-74.0)	.448
2 y	84.3 ± 21.6 (28.0-100.0)	79.6 ± 21.2 (35.0-100.0)	82.3 ± 21.4 (28.0-100.0)	.404
10 y	72.9 ± 23.5 (4.3-100.0)	79.9 ± 23.4 (16.2-99.1)	76.0 ± 23.6 (4.3-100.0)	.020
ASES				
Baseline	48.4 ± 17.4 (16.0-75.0)	57.8 ± 17.4 (12.0-88.0)	52.7 ± 17.9 (12.0-88.0)	.022
2 y	89.1 ± 16.2 (43.0-100.0)	89.2 ± 13.4 (55.0-100.0)	89.2 ± 15.0 (55.0-100.0)	.974
10 y	80.4 ± 23.6 (15.0-100.0)	83.0 ± 25.1 (10.0-100.0)	81.6 ± 24.1 (10.0-100.0)	.675
Constant				
Baseline	56.1 ± 14.0 (23.0-77.0)	60.4 ± 18.1 (16.0-92.0)	58.1 ± 16.0 (16.0-92.0)	.250
2 y	86.5 ± 14.1 (32.0-100.0)	85.9 ± 13.3 (52.0-100.0)	86.3 ± 13.7 (32.0-100.0)	.855
10 y	77.2 ± 16.7 (32.0-98.0)	71.3 ± 25.6 (0.0-98.0)	74.4 ± 21.4 (0.0-98.0)	.348
Strength, kg				
Baseline	4.8 ± 1.8 (1.8-9.1)	4.8 ± 3.2 (0.9-13.6)	4.8 ± 2.5 (0.9-13.6)	.99
2 y	7.9 ± 2.6 (3.6-11.3)	7.3 ± 3.6 (1.8-11.8)	7.7 ± 3.0 (1.8-11.8)	.56
10 y	7.1 ± 2.1 (0.9-11.3)	6.9 ± 2.7 (2.3-11.3)	7.0 ± 3.0 (0.9-11.3)	.87

a Data are shown as mean ± SD (95% CI). ASES, American Shoulder and Elbow Surgeons; WORC, Western Ontario Rotator Cuff Index.

A comparison of the within-group change in WORC scores using mixed-effects linear regression from preoperatively (baseline) to 10-year follow-up showed a decline in the double-row (−40.9 [95% CI, −49.5 to −32.1]; *P* < .0001) and single-row (−36.0 [95% CI, −44.8 to −29.0]; *P* < .0001) groups.

A comparison of WORC scores from 2- to 10-year follow-up demonstrated a small difference in the double-row group (–0.2 [95% CI, −9.6 to 9.2]; *P* = .963), but a significant decrease in the single-row group (11.5 [95% CI, 3.3 to 19.7]; *P* = .006) was observed (Table 3). Similarly, there was a significant decline in ASES scores in the single-row group between 2- and 10-year follow-up (*P* = .029), which was not seen in the double-row group (*P* = .195). Constant scores for both the single- and double-row groups declined significantly between 2 and 10 years. Strength scores also decreased from 2 to 10 years, although this was not statistically significant. Within-group changes in scores are summarized in Table 3.

**Table 3:** Within-Group Change in Outcome Scores Over Time^*a*^

	Mean Change^*b*^	95% CI	*P* Value
WORC			
Double row			
Baseline to 2 y	−40.6	−50.1 to −31.1	<.0001
Baseline to 10 y	−40.9	−49.5 to −32.1	<.0001
2 to 10 y	−0.2	−9.6 to 9.2	.963
Single row			
Baseline to 2 y	−48.5	−56.8 to −40.2	<.0001
Baseline to 10 y	−36.0	−44.8 to −29.0	<.0001
2 to 10 y	11.5	3.3 to 19.7	.006
ASES			
Double row			
Baseline to 2 y	−30.9	−39.9 to −21.8	<.0001
Baseline to 10 y	−24.6	−33.3 to −15.9	<.0001
2 to 10 y	6.2	−3.2 to 15.6	.195
Single row			
Baseline to 2 y	−41.0	−48.8 to −33.1	<.0001
Baseline to 10 y	−31.8	−39.8 to −23.7	<.0001
2 to 10 y	9.2	0.9 to 17.5	.029
Constant			
Double row			
Baseline to 2 y	−25.4	−33.4 to −17.4	<.0001
Baseline to 10 y	−10.0	−19.1 to −2.7	.009
2 to 10 y	14.4	5.6 to 23.3	.001
Single row			
Baseline to 2 y	−30.9	−37.8 to −23.9	<.001
Baseline to 10 y	−21.4	−29.2 to −13.6	<.0001
2 to 10 y	9.5	1.4 to 17.5	.020
Strength			
Double row			
Baseline to 2 y	−2.7	−3.8 to −1.5	<.0001
Baseline to 10 y	−1.7	−2.9 to −0.4	.007
2 to 10 y	0.9	−0.4 to 2.3	.15
Single row			
Baseline to 2 y	−2.8	−3.8 to −1.8	<.0001
Baseline to 10 y	−2.6	−3.7 to −1.4	<.0001
2 to 10 y	0.2	−1.0 to 1.4	.748

a ASES, American Shoulder and Elbow Surgeons; WORC, Western Ontario Rotator Cuff Index.

b Negative value indicates an increase in the score.

Between-group changes in scores are summarized in Table 4. The between-group comparison of the change in scores from baseline to 10 years for the WORC demonstrated a difference of 3.9 (95% CI, −7.8 to 15.6; *P* = .510). However, the comparison of between-group changes in scores from 2 to 10 years showed a difference of 11.7 (95% CI, –0.7 to 24.3) in favor of the double-row group, which trended toward statistical significance (*P* = .065). The change in ASES scores was not significantly different between groups, and no significant differences were observed between groups in the Constant score or strength.

**Table 4:** Between-Group Change in Outcome Scores Over Time^*a*^

	Mean Change^*b*^	95% CI	*P* Value
WORC			
Double row from baseline to 2 y vs single row from baseline to 2 y	−7.8	−20.4 to 4.7	.219
Double row from baseline to 10 y vs single row from baseline to 10 y	3.9	−7.8 to 15.6	.510
Double row from 2 to 10 y vs single row from 2 to 10 y	11.7	−0.7 to 24.3	.065
ASES			
Double row from baseline to 2 y vs single row from baseline to 2 y	−10.1	−22.0 to 1.8	.097
Double row from baseline to 10 y vs single row from baseline to 10 y	−7.1	−18.9 to 4.7	.237
Double row from 2 to 10 y vs single row from 2 to 10 y	2.9	−9.5 to 15.5	.638
Constant			
Double row from baseline to 2 y vs single row from baseline to 2 y	−5.4	−16.1 to 5.1	.307
Double row from baseline to 10 y vs single row from baseline to 10 y	−10.4	−21.7 to 0.8	.069
Double row from 2 to 10 y vs single row from 2 to 10 y	−4.9	−16.9 to 6.9	.409
Strength			
Double row from baseline to 2 y vs single row from baseline to 2 y	−0.1	−1.6 to 1.4	.868
Double row from baseline to 10 y vs single row from baseline to 10 y	−0.9	−2.6 to 0.7	.29
Double row from 2 to 10 y vs single row from 2 to 10 y	−0.7	−2.6 to 1.0	.39

a ASES, American Shoulder and Elbow Surgeons; WORC, Western Ontario Rotator Cuff Index.

b Positive value indicates that the outcome is in favor of the double-row group.

Many patients were unwilling to return for tendon imaging by ultrasound. A total of 30 patients (18 in single-row group and 12 in double-row group) underwent ultrasound to evaluate the integrity of rotator cuff repair at 10 years postoperatively. Overall, 14 patients in the single-row group (77%) had an intact tendon compared with 7 patients in the double-row group (58%) (*P* = .418). There were 3 patients with a previously intact tendon at 2 years who developed a full-thickness tear at 10 years (1 in single-row group and 2 in double-row group). One patient from each study group underwent revision surgery after the 2-year time point.

## Discussion

There is a paucity of long-term outcome data in the literature regarding the single- versus double-row technique in arthroscopic rotator cuff repair.^14,27^ To our knowledge, no randomized controlled trials have reported the long-term effects of these techniques on clinical and anatomic results. At 10 years postoperatively, we found that the WORC score for the double-row group was statistically higher than that for the single-row group in patients who underwent arthroscopic rotator cuff repair, but this difference was unlikely to be clinically important. Furthermore, no other significant differences were detected between groups for secondary outcomes including the ASES score, Constant score, or strength. Therefore, the original hypothesis of superior quality of life and functional outcomes with double-row fixation compared with single-row fixation was not supported. The mixed-effects linear regression analyses of the change in scores between the single- and double-row groups did not demonstrate any differences between baseline and 10 years. However, between 2- and 10-year follow-up, both WORC and ASES scores were maintained in the double-row group but declined significantly in the single-row group. The Constant score declined in both the single- and the double-row groups between 2- and 10-year follow-up.

There have been varying reports in the literature regarding the short-term outcomes of single- versus double-row arthroscopic rotator cuff repair. Essentially, 7 randomized controlled trials have been published to investigate the short-term results of rotator cuff repair.^3,4,9,11,13,19,20^ With a mean follow-up duration of 23.2 months, no detectable significant differences in ASES, University of California, Los Angeles (UCLA), and Constant scores were found between single- and double-row repair. However, there was a trend toward an increased risk of retears with single-row repair compared with double-row repair.

A few other studies have examined the outcomes of arthroscopic rotator cuff repair at medium- to long-term follow-up. Marrero et al^22^ published the results of a case series consisting of 24 patients with a minimum follow-up of 9 years, reporting a mean UCLA score of 31.8, with 87.7% of patients having excellent and good outcomes. Comparable findings were also recorded by Miyazaki et al^26^ for 35 cases of arthroscopic repair of massive rotator cuff tears; they reported good functional results (UCLA score, 31.31), and 91% of the patients continued to present good and excellent results (40% excellent and 51% good) at a minimum of 9 years postoperatively. Boorman et al^2^ reported that with nonoperative treatment of rotator cuff tears, approximately 75% remained successfully treated at 5 years without surgery. However, the functional status of patients treated nonoperatively beyond 5 years has not been reported, to our knowledge.

Long-term outcomes of rotator cuff repair in the context of comparative single- versus double-row techniques have been scarce in the literature. One cohort study by Plachel et al^27^ attempted to elucidate the continuing effects of arthroscopic rotator cuff repair by comparing the functional and radiological outcomes between the 2 techniques. No significant difference was found between the fixation techniques with regard to WORC and Constant scores at a minimum of 10 years after surgery. This finding was in keeping with our results. Similarly, the overall WORC score at the final follow-up in their study decreased slightly from 95% ± 7% to 87% ± 18% (*P* < .05). Interestingly, although primary (Constant) and secondary (WORC, ASES, Subjective Shoulder Value, and Simple Shoulder Test) outcomes showed no difference between the 2 groups, the retear rate was higher with single-row repair (55%) compared with double-row repair (33%) (*P* = .370). The favorable results might be indicative of superior healing rates with double-row repair secondary to enhanced biomechanical properties.

Other studies have demonstrated an association between rotator cuff integrity and clinical outcomes. Randelli et al^29^ reported a retear rate of 52% in a group of 102 patients who underwent single-row arthroscopic rotator cuff repair. Patients with healed rotator cuff tendons demonstrated superior functional scores, satisfaction, range of motion, and flexion strength; lower grades of osteoarthritis; and higher acromiohumeral distances. In a retrospective study of 30 patients, Heuberer et al^14^ observed that the Constant total score and Constant strength subscore were significantly better at 10 years postoperatively in patients with intact tendons compared with patients with retorn tendons.

The results of our study were in line with recent investigations addressing long-term results after arthroscopic rotator cuff repair with the single- versus double-row technique. A statistical (but not clinical) difference was seen in favor of double-row fixation with regard to our primary outcome. Mixed-effects linear regression did not demonstrate a difference in either the within- or the between-group change in scores from baseline to 10 years. However, although the single-row group had a higher WORC score at 2 years compared with the double-row group (84.3 ± 21.6 vs 79.6 ± 21.2, respectively), the analysis of the change in scores from 2 to 10 years demonstrated that patients treated with double-row repair preserved the functional gains in 2-year WORC and ASES scores through to 10-year follow-up, whereas a significant functional decline was seen in the single-row group after 2 years. Yet, this difference was not observed in the within- or between-group analysis of Constant or strength scores. The reasons behind the decline in functional scores are not clear. It is possible that further tendon degeneration mediated by stem cell senescence occurred over time. If this is the case, it is conceivable that initial double-row fixation provided some protection against loss of function, possibly related to an increased surface area from initial tendon/bone healing compared with single-row fixation, but further studies are required to verify this hypothesis.

Our data have shown that the majority of retears occurred before 2 years postoperatively, as the healing rate was 67% in the single-row group compared with 78% in the double-row group (*P* = .254).^20^ By 10-year follow-up, an additional 10% of patients sustained tears. These findings were in accordance with Heuberer et al,^14^ who demonstrated that only 17% of tendons deteriorated from 2 years to long-term follow-up. Similarly, Kluger et al^18^ reported an overall retear rate of 33%, with 86% of the tears occurring in the first 2 years, and an additional 4.7% of tears reruptured between the second and fifth years.

The present study has some limitations. There was a loss to follow-up rate for the primary outcome measure of 15% at 10 years. However, this loss was considerably lower than in previously published studies^18,27^ and the characteristics of the randomized controlled trial population remained representative, as none of the baseline characteristics differed meaningfully between all participants and those who remained in the trial at 10 years. Moreover, 67% of the patients did not agree to return for postoperative imaging, and therefore, the imaging results must similarly be viewed with caution. Ultrasound instead of magnetic resonance imaging was used to assess tendon integrity. However, as both modalities have been validated for high sensitivity to detect rotator cuff tears,^6,8^ including in the postoperative setting,^28^ we do not believe that this negatively affects the quality of the data.

## Conclusion

Statistically superior WORC scores were seen in favor of double-row fixation at 10-year follow-up, although this was unlikely to be a clinically important difference. However, double-row fixation led to the preservation of joint function out to 10 years, while single-row fixation demonstrated a clinically important functional decline on 2 of the 3 outcome tools used. Future studies should focus on whether these results occur in other populations and in determining the long-term results with regard to healing rates.
